# Niobium-titanium (Nb-Ti) superconducting joints for persistent-mode operation

**DOI:** 10.1038/s41598-019-50549-7

**Published:** 2019-10-03

**Authors:** Dipak Patel, Su-Hun Kim, Wenbin Qiu, Minoru Maeda, Akiyoshi Matsumoto, Gen Nishijima, Hiroaki Kumakura, Seyong Choi, Jung Ho Kim

**Affiliations:** 1 Institute for Superconducting and Electronic Materials, Australian Institute for Innovative Materials, University of Wollongong, North Wollongong, New South Wales 2500 Australia; 2 National Institute for Materials Science (NIMS), 1-2-1 Sengen, Tsukuba, Ibaraki 305-0047 Japan; 3 Department of Electrical Engineering, Kyungpook National University, Daegu, 41566 Republic of Korea; 4 Department of Electrical Engineering, Kangwon National University, Kangwon, 25913 Republic of Korea

**Keywords:** Superconducting properties and materials, Ceramics

## Abstract

Superconducting joints are essential for persistent-mode operation in a superconducting magnet system to produce an ultra-stable magnetic field. Herein, we report rationally designed niobium-titanium (Nb-Ti) superconducting joints and their evaluation results in detail. For practical applications, superconducting joints were fabricated by using a solder matrix replacement method with two types of lead-bismuth (Pb-Bi) solder, including Pb_42_Bi_58_ as a new composition. All the joints attained a critical current of >200 A below 1.43 T at 4.2 K. Our optimized superconducting joining method was tested in a closed-loop coil, obtaining a total circuit resistance of 3.25 × 10^−14^ Ω at 4.2 K in self-field. Finally, persistent-mode operation was demonstrated in an Nb-Ti solenoid coil with a persistent-current switch. This work will pave the way to developing high-performance Nb-Ti superconducting joints for practical applications.

## Introduction

Superconducting magnets are used to produce a strong magnetic field in a compact system, which otherwise cannot be attained using conventional copper (Cu) based magnets. Niobium-titanium (Nb-Ti) with a critical temperature (*T*_c_) of 9.2 K is usually used to fabricate superconducting magnets for various practical applications. One of the distinct features of Nb-Ti that makes it ideal for most commercial applications is the possibility to produce reliable “superconducting joints”. The superconducting joints allow the Nb-Ti magnet to operate in the persistent-mode to attain an ultra-stable (long-term drift rate of magnetic field on the order of 0.1 ppm∙h^−1^) magnetic field^1^.

To achieve persistent-mode operation, two ends of an Nb-Ti superconducting magnet should be connected with a persistent-current switch (PCS) using superconducting joints without any electrical loss. To date, various methods have been reported to fabricate the superconducting joints of Nb-Ti conductors, including solder matrix replacement^2–6^, ultrasonic welding^7^, diffusion welding^8^, cold-pressing^9,10^, and spot welding^10,11^. Among these methods, the solder matrix replacement method with lead-bismuth (Pb-Bi) solder, which is superconducting at 4.2 K, is a common choice in industrial use to fabricate Nb-Ti joints from the viewpoint of reliability^11^. The first high-performance Nb-Ti joints using the solder matrix replacement method were reported by Thornton in 1986^2^. A closed-loop with a single joint (fabricated in open air) achieved a critical current density (*J*_c_) as high as 143 kA·cm^−2^ in self-field at 4.2 K. Swenson *et al*. fabricated several Nb-Ti joints using the Thornton method and measured their resistance by the standard four-probe method^3^. They achieved joint resistances of <1 × 10^−11^ Ω in 1.5 T at 4.2 K. Cheng *et al*. also reported results on several Nb-Ti joints for a 400 MHz nuclear magnetic resonance magnet^4^. One of their joints in a closed-loop achieved a critical current (*I*_c_) of 89.5 A and joint resistance of 1.8 × 10^−13^ Ω in 1 T at 4.2 K. In addition, Liu *et al*. fabricated and tested Nb-Ti joints for a 7 T animal magnetic resonance imaging (MRI) magnet^5^. Their joints fabricated using 1.5 × 1 mm^2^ Nb-Ti/Cu wire achieved an *I*_c_ of 1,160 A and a resistance of 1.5 × 10^−14^ Ω in 0.6 T at 4.2 K. More recently, in 2015, Motomune *et al*. studied current paths in Nb-Ti joints fabricated using the solder matrix replacement method^6^. Despite that Nb-Ti superconducting joints are routinely fabricated in the MRI industry and are a critical component of MRI magnets, research on superconducting joining processes for multifilamentary Nb-Ti conductors has been limited.

In response, in this work, two types of Pb-Bi solder were evaluated as potential candidates for Nb-Ti superconducting joints in terms of their *J*_c_ and *T*_c_. The optimal etching time for the Cu matrix with tin (Sn) and Sn with Pb-Bi was then evaluated by scanning electron microscopy (SEM). Based on these findings, superconducting joints were fabricated in a partially inert atmosphere to avoid oxidation and characterized under different magnetic fields at 4.2 K. To precisely estimate the joint resistance, a single-turn Nb-Ti closed-loop coil was fabricated and evaluated by using the field-decay measurement method. The suitability of the newly developed superconducting joints for a persistent-mode magnet was verified by demonstrating persistent-mode operation in a prototype Nb-Ti magnet.

## Results and Discussion

**Figure 1:**
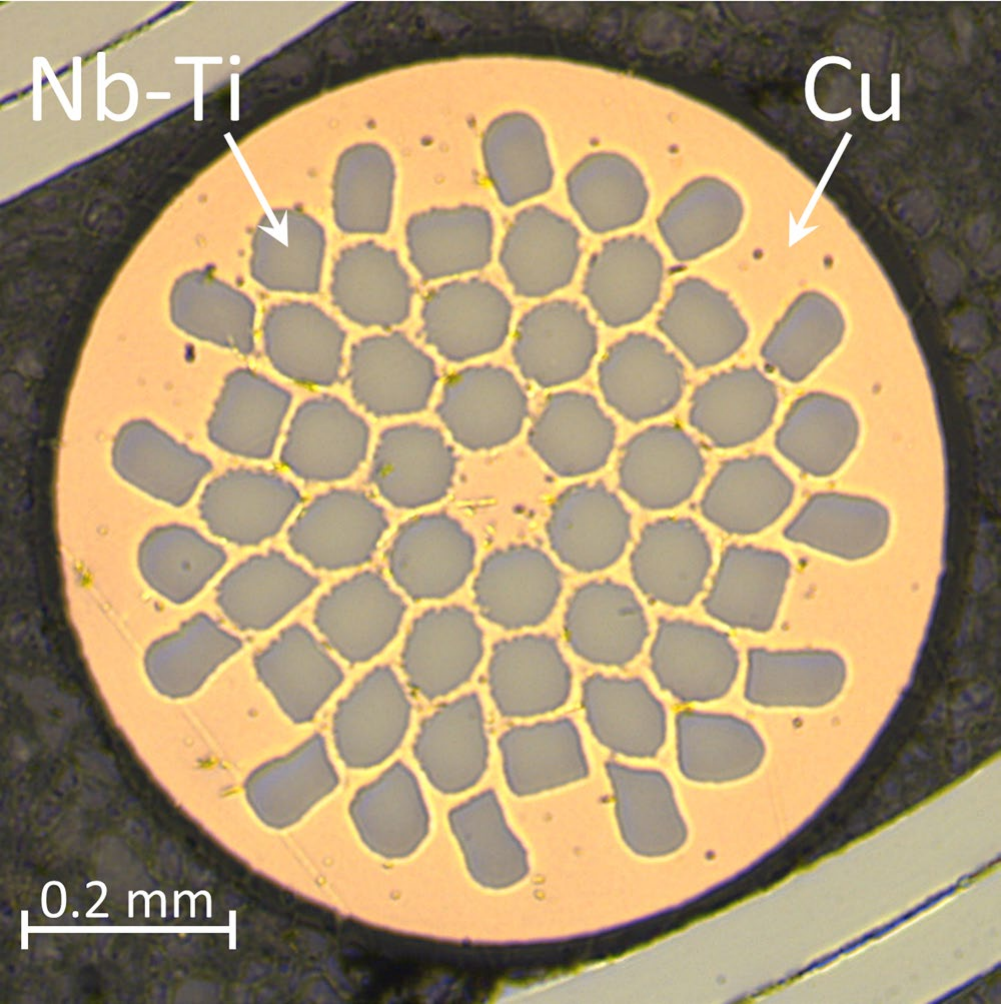
Cross-sectional image of the Nb-Ti wire.

The multifilament Nb-Ti wire for this work was supplied by Luvata^12^. Figure 1 shows a cross-sectional image of the Nb-Ti wire and its specifications are listed in Table S1 in the Supporting Information.

**Figure 2:**
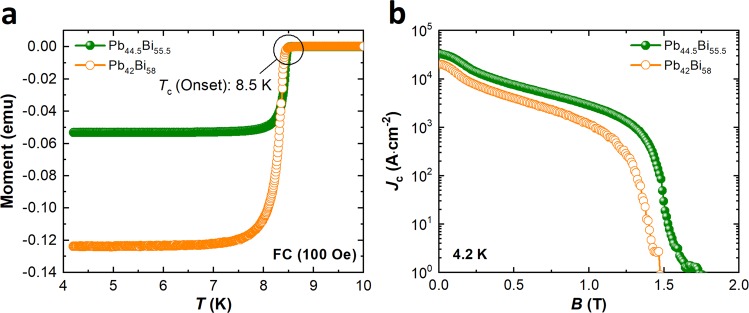
(**a**) Magnetic moment of the solders versus temperature under field cooling (FC) in self-field; (**b**) critical current density versus magnetic field characteristics measured using a physical property measurement system (PPMS).

For our superconducting joints, we selected two types of Pb-Bi alloys as solder^13^. In the first step, we checked the element composition using energy dispersive X-ray spectroscopy (EDS) because the superconductivity of Pb-Bi at 4.2 K is very sensitive to its composition and the age of the solder from the time of its manufecturing^14,15^. This is because at room temperature Bi precipitate out of superconducting ${\epsilon }$ phase in these Pb-Bi alloys due to solid state diffusion^15,16^. In particular, a slight change in the alloy composition can result in different electrical properties^14^. Since both the solders were procured from the commercial supplier, the exact age of the solders was not known^13^. Contrary to company specifications, we found deficient Pb in the two types of solder, as can be seen in Table 1. This is probably because the Pb is much more likely to be volatile, even at low temperature, during melting with Bi. Brittles also found deficient Pb content in their Pb-Bi solder^16^. In the second step, we evaluated the *T*_c_ and magnetic *J*_c_ of the two Pb-Bi solders, as can be seen in Fig. 2. The *T*_c_ values for the two solders were estimated to be 8.5 K, with only a negligible difference. The two solders with different compositions (Pb_44.5_Bi_55.5_ and Pb_42_Bi_58_) showed different *J*_c_ performance. For example, the magnetic *J*_c_ for Pb_44.5_Bi_55.5_ was larger than that for Pb_42_Bi_58_ under a magnetic field. According to the binary Pb-Bi phase diagram (see ref.^15^), at Bi concentration from 55.5 to 58.0 wt.%, the alloys consist of superconducting ${\epsilon }$ and non-superconducting Bi phases. The higher magnetic *J*_c_ obtained in the Pb_44.5_Bi_55.5_ can be attributed to improved flux pinning at Bi precipitates^2^. However, beyond certain Bi concentration in the Pb-Bi alloys, Bi precipitates can act as impurities, which would have eventually reduced *J*_c_ of the Pb_42_Bi_58_ under magnetic field compared with the Pb_44.5_Bi_55.5_^14^. A comparison of the magnetic *J*_c_ values reported thus far is summarized in Table S2. As can be seen in the table, the commercial Pb_44.5_Bi_55.5_ used in this work showed a relatively high *J*_c_ compared to earlier reported results^13^.

**Table 1:** Specifications of the Pb-Bi alloys.

Commercial material	Measured by EDS	Melting temperature (°C)
Pb_44.5_Bi_55.5_	Pb_42.9_Bi_57.1_	123.9
Pb_42_Bi_58_	Pb_40.8_Bi_59.2_	123.9–126.1

**Figure 3:**
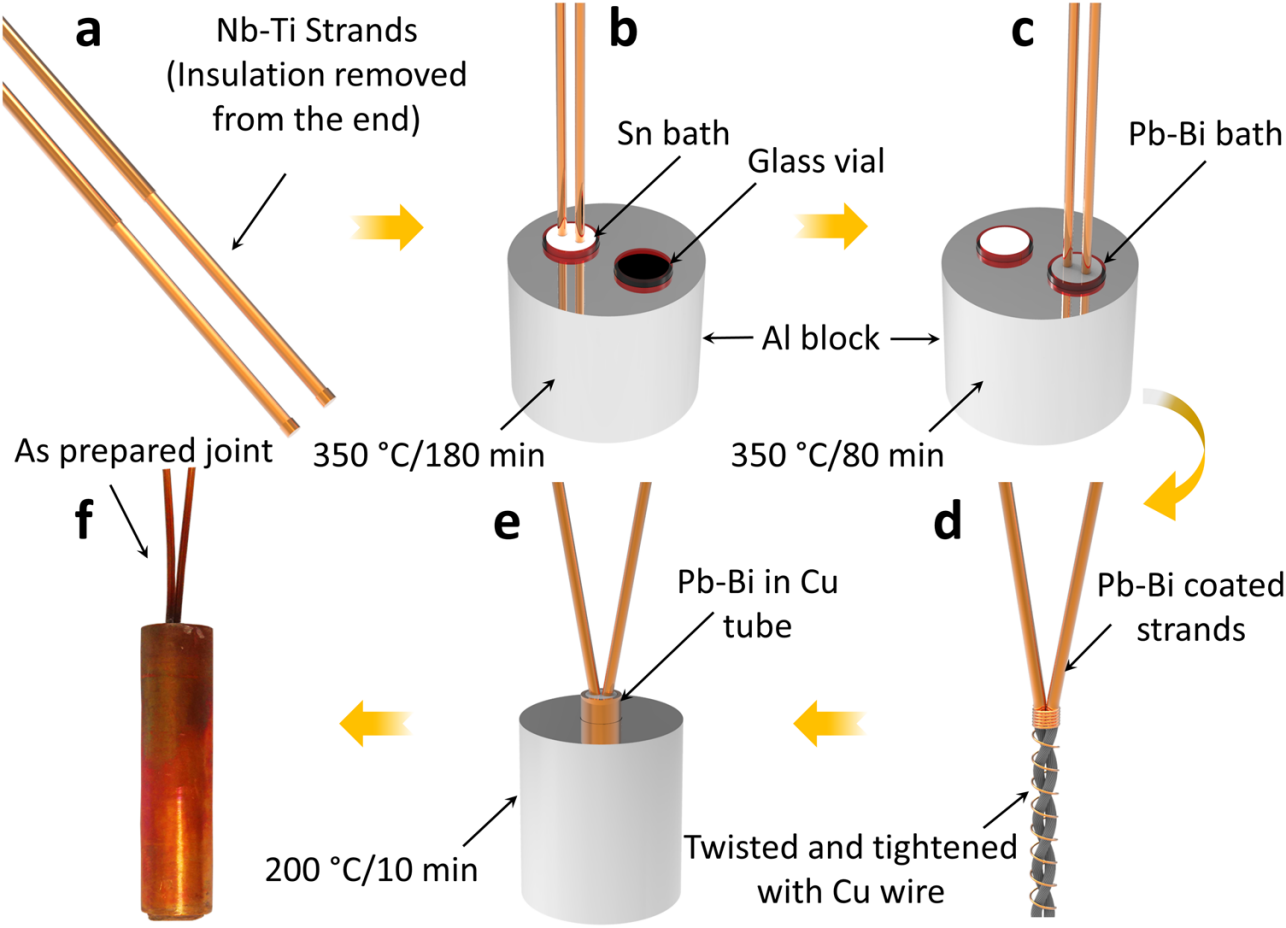
Joint fabrication process for multifilamentary Nb-Ti conductors using the solder matrix replacement method.

Figure 3 shows the joint fabrication process for multifilament Nb-Ti conductors using the solder matrix replacement method. First, as shown in Fig. 3a, the insulation material (polyvinyl acetate (PVA), Formvar^®^) of the wires was removed using abrasive paper. In a 45 mm length, only 43 mm of insulation was removed, and 2 mm of Formvar insulation was kept intact at the ends for handling the fine Nb-Ti filaments after etching the Cu or Cu-Ni (Nickel) matrix. After removing the insulation and cleaning using acetone, the wires were next immersed in a molten Sn bath at 350 °C for 180 min, as can be seen in Fig. 3b^17^. The Sn coated Nb-Ti wires were then immersed in a molten Pb-Bi bath at 350 °C for 80 min (Fig. 3c)^13^. The Pb-Bi coated wires were thereafter removed from the bath and allowed to naturally cool in open air. Subsequently, the insulated ends of the Pb-Bi coated Nb-Ti wires were cut off. The two wires were aligned, twisted, and tightened with fine Cu wire in open air to enhance their electrical and mechanical properties, as shown in Fig. 3d. Finally, the twisted wires were again immersed in a Cu tube filled with molten Pb-Bi at 200 °C for 10 min (Fig. 3e). The dimensions of the Cu tube were 5.7 mm, 8 mm, and 53 mm (inner diameter (I.D.), outer diameter (O.D.), and height, respectively). After 10 min, heating was stopped, and the joint was allowed to naturally cool to room temperature (Fig. 3f). To minimize oxidation, pure argon was continuously flowed on top of the baths while the wires were inserted and taken out. Occasionally, the Sn and Pb-Bi baths were stirred using the immersed Nb-Ti wires.

To technically determine the appropriate etching time of the Cu matrix in the wire, a total of six wires were prepared (Fig. 3a) and immersed in molten Sn at 350 °C, as shown in Fig. 3b. One wire was removed from the bath at 30 min intervals (30, 60, 90, 120, 150, and 180 min) up to 180 min. Each wire was cut from three different locations (top, middle, and bottom). Elemental mapping of each cross-section was carried out using SEM. After an etching time of 150 min, some Cu still existed in the bottom part of the wire. Most of the Cu was thoroughly replaced by Sn after 180 min.

In the next step to determine the time required for replacement of Sn by Pb-Bi, a total of five wires were prepared and immersed in molten Sn at 350 °C for 180 min. The Sn coated Nb-Ti wires were then immersed in molten Pb_42_Bi_58_ at 350 °C. One wire was removed from the bath at 20 min intervals (20, 40, 60, 80, and 100 min) up to 100 min. Elemental mapping of each cross-section was carried out using SEM. Most of the Sn was replaced by Pb-Bi after 60 min of etching time. Further close observation between the edges of two Nb-Ti filaments revealed, however, some residual Sn on the edges, as shown in Fig. S1a. To determine the thickness of the Sn layer at the edges of the Nb-Ti filaments, elemental line mapping was carried out between the filaments in the samples etched for 60, 80, and 100 min in Pb-Bi. As can be seen in Fig. S1b, the Sn layer thickness in all three samples was quite similar. This indicates that longer etching time in Pb-Bi could not remove the thin Sn layer from the edges of the Nb-Ti filaments. To allow slightly more time for etching Sn, etching time of 80 min was chosen for Pb-Bi in our joint fabrication process.

**Figure 4:**
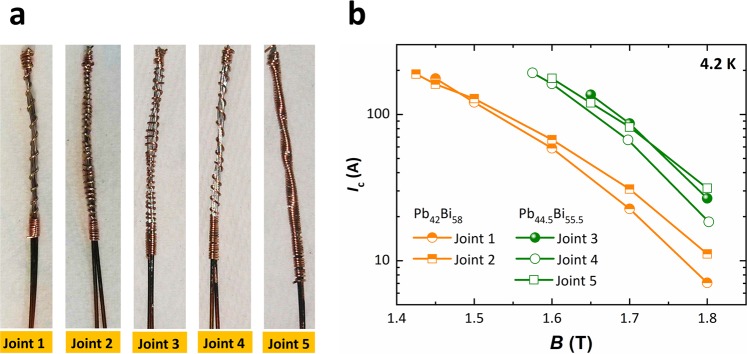
(**a**) Different types of Cu wire binding on joints 1 to 5, (**b**) critical current versus magnetic field characteristics of the joints.

After determining the optimal joint fabrication conditions, five specimens were fabricated with different Cu binding configurations, as shown in Fig. 4a. Joints 1 and 2 were fabricated using Pb_42_Bi_58_, whereas joints 3, 4, and 5 were fabricated using Pb_44.5_Bi_55.5_. Figure 4b shows the magnetic field dependence of *I*_c_ of the Nb-Ti joints in different magnetic fields up to 1.8 T at 4.2 K. As shown in the figure, joints 1 and 2 presented an *I*_c_ > 200 A in a field <1.43 T at 4.2 K, and thus the *I*_c_ results from 1.4 T to 1.8 T are shown. Likewise, joints 3, 4, and 5 reached an *I*_c_ > 200 A in a field < 1.58 T at 4.2 K. The joints made using Pb_42_Bi_58_ were expected to result in poor electrical performance, as shown in Fig. 2b. The poor performance of joint 1 and joint 4 compared to the other joints in their group indicate that loose Cu wire binding (see Fig. 4a) in the joint is not favorable for achieving high performance. Thus, the Cu wire binding should be reasonably tight. Joint 3 reached its highest *I*_c_ of 136 A in 1.65 T at 4.2 K, whereas joint 5 reached its highest *I*_c_ of 31 A in 1.8 T at 4.2 K. The electric field versus current characteristics of joint 5 in different magnetic fields at 4.2 K is shown in Fig. S2. As can be seen in the figure, the superconducting to normal transition up to 0.1 µV∙cm^−1^ criterion appears not very sharp but rather smooth. The magnetic *J*_c_ values of Pb_42_Bi_58_ and Pb_44.5_Bi_55.5_ dropped to 1 A·cm^−2^ at 1.47 T and 1.66 T, respectively, at 4.2 K. It is expected that direct filament to filament contact between two Nb-Ti strands would have been established, and thereby direct current transfer would take place between the filaments. The Nb-Ti joint performance achieved in this work using Pb_44.5_Bi_55.5_ is acceptable for application of the joints in a persistent-mode magnet.

**Figure 5:**
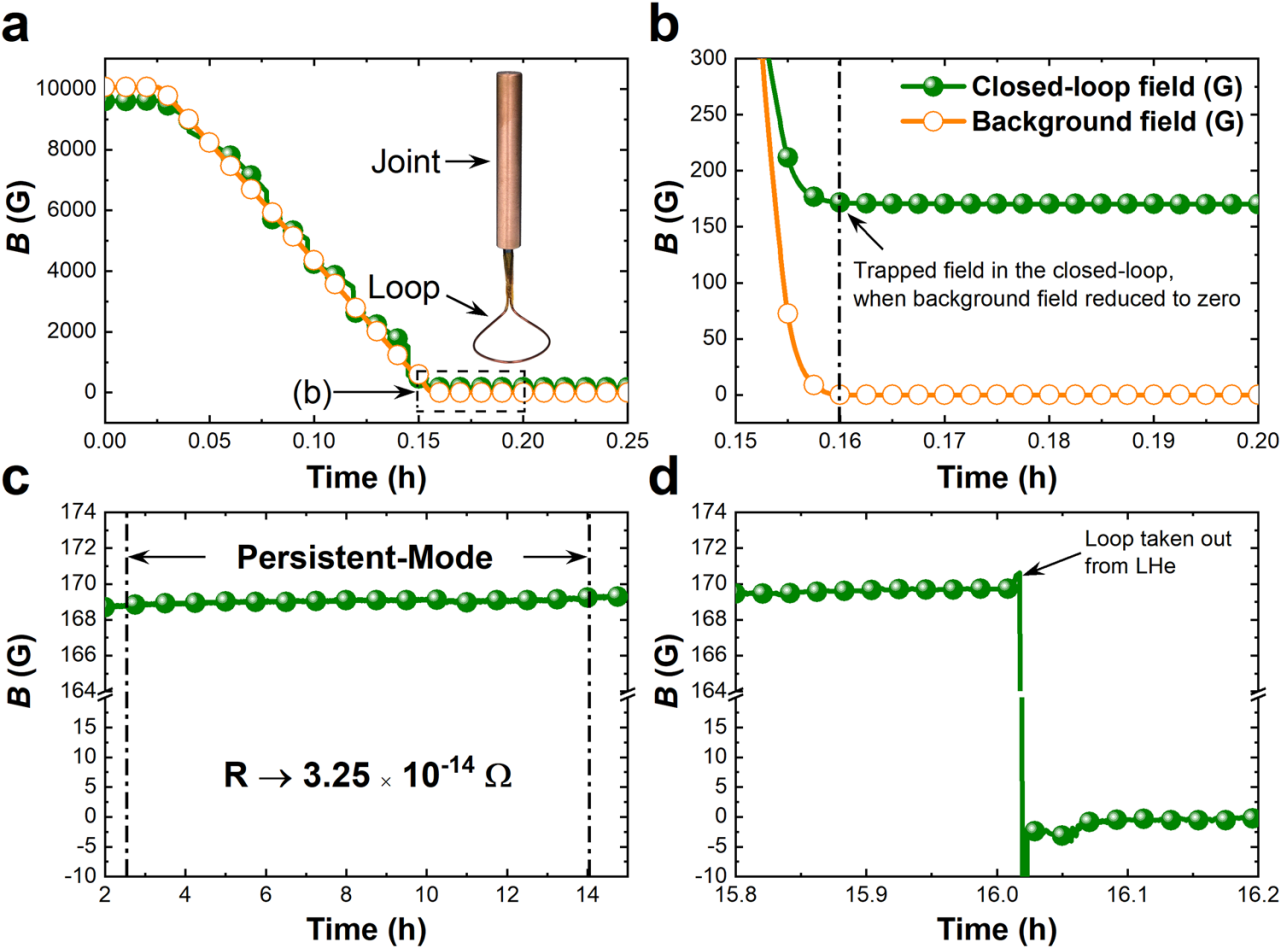
Magnetic field with respect to time characteristics for a closed-loop coil containing the optimized joint: (**a**) while discharging background magnetic field, (**b**) trapped field in the closed-loop when the background field was reduced to zero, (**c**) while the closed-loop was in persistent-mode (after initial stabilization), and (**d**) when the closed-loop coil was taken out of LHe. The inset of (**a**) is the Nb-Ti closed-loop coil together with the joint. The legends in (**a**–**d**) are the same.

Following the optimization of the joint fabrication process using Pb_42.5_Bi_55.5_, a single-turn Nb-Ti closed-loop coil was fabricated, as shown in the inset of Fig. 5(a), and the joint resistance was estimated by field-decay measurements. The specifications of the closed-loop coil are listed in Table 2. Figure 5(a) shows the magnetic field with respect to time characteristics for the coil while discharging the background field. As can be seen in Fig. 5(b), when the background field was reduced to zero, the trapped field in the closed-loop coil was 170.6 gauss (G), which was equivalent to 228.6 A. The trapped field was allowed to stabilize for about 2 hours (h). At 0.574 µH inductance of the coil and 0.43 G field variation in 12.5 hours (Fig. 5(c)), the calculated circuit resistance according to field-decay in the *L* - *R* circuit was 3.25 × 10^−14^ Ω^18–20^. This circuit resistance meets the technical requirement for a persistent-mode superconducting magnet system. As shown in Fig. 5(d), when the test probe along with the coil was removed from the liquid helium (LHe) bath, the trapped field decayed to zero.

**Table 2:** Specifications of the single-turn Nb-Ti closed-loop coil.

Parameters	Specifications
Strand	Nb-Ti/Cu
Strand diameter insulated/bare (mm)	0.77/0.72
Solder used in the joint	Pb_42.5_Bi_55.5_
Coil I.D. (mm)	24
**Computed using FE analysis**	
Inductance (µH)	0.574
Trapped field at the Hall sensor (G)	170.6
Initial trapped current (A)	228.6

**Figure 6:**
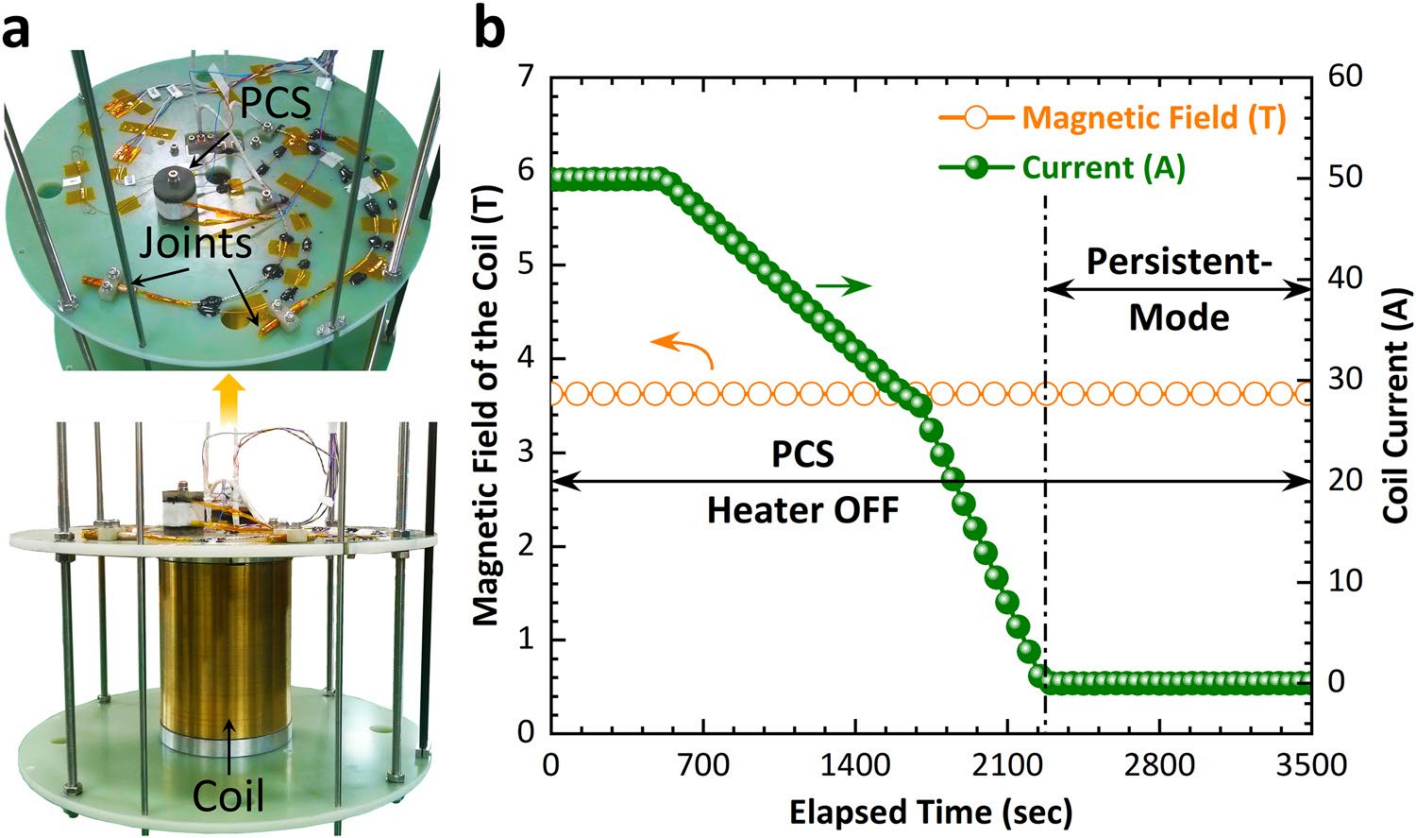
(**a**) Digital images of the Nb-Ti solenoid coil along with the joints and PCS, (**b**) magnetic field of the coil and coil current versus elapsed time during the persistent-mode demonstration.

Finally, a prototype Nb-Ti coil was fabricated to demonstrate persistent-mode operation. The specifications of the Nb-Ti coil and PCS are summarized in Table 3. There were two joints between the Nb-Ti coil and the PCS, as shown in Fig. 6a. Our optimized joining process was the same as that employed for joint 3. The PCS was installed far from the main Nb-Ti coil, since the generated field of 3.6 T must not obstruct the PCS. In our empirical environment, we tested the Nb-Ti coil in persistent-mode at 50 A (central field ~3.6 T) in an LHe bath. As can be seen in Fig. 6b, the Nb-Ti coil was successfully operated in the persistent-mode for about 1,235 s without any noticeable decay in the coil current.

**Table 3:** Specifications of the Nb-Ti coil and PCS.

Parameters	Specifications
Coil type	Solenoid
Winding method	Wet winding
Strand	Nb-Ti/Cu
Strand diameter insulated/bare (mm)	0.9/0.85
Coil I.D./O.D./Height (mm)	84/152/200
Number of turns	12,950
Inductance (H)	7.2
Operating current (A)	97
Magnetic field at the center (T)	7
PCS I.D./O.D./Height (mm)	30/37.2/26
PCS (number of turns)	112
PCS strand	Same as coil
PCS heater	Nichrome wire

## Conclusions

We have presented a superconducting joining process for a multifilament Nb-Ti conductor using a solder matrix replacement method and test results on the superconducting joints. First, EDS mapping of two Pb-Bi solders, Pb_44.5_Bi_55.5_ and Pb_42_Bi_58_, was carried out to confirm their compositions, which may be related to their superconducting properties. Both solders showed up to 1.6% Pb deficiency. The *T*_c_ (onset) of 8.5 K was measured for both solders, whereas the magnetic *J*_c_ of Pb_44.5_Bi_55.5_ and Pb_42_Bi_58_ was found to be 2.9 × 10^3^ A·cm^−2^ and 1.19 × 10^3^ A·cm^−2^, respectively, in 1 T at 4.2 K. The *J*_c_ of Pb_44.5_Bi_55.5_ represented the highest performance reported to date. One of the best joints fabricated using Pb_44.5_Bi_55.5_ showed an *I*_c_ of 136 A in 1.65 T at 4.2 K. The poor performance of the joints made using Pb_42_Bi_58_ compared with Pb_44.5_Bi_55.5_ was consistent with the magnetic measurement results. We also reported that loose Cu wire binding in the joint was not favorable in terms of achieving high joint performance. A single-turn Nb-Ti closed-loop coil was fabricated along with the joint to precisely estimate the joint resistance using the field-decay measurement. The measured total circuit resistance was 3.25 × 10^−14^ Ω in self-field at 4.2 K, which meets the technical requirements for persistent-mode operation. Finally, the persistent-mode operation of a Nb-Ti solenoid coil having two joints and a PCS was demonstrated. The systematic study and results presented in this work on the Nb-Ti superconducting joining process will pave the way for developing high-performance Nb-Ti superconducting joints.

## Experimental

The elemental composition of Pb-Bi alloys was evaluated using the EDS capability of a JEOL low-vacuum SEM. To observe the longitudinal cross-section and to conduct EDS using the SEM, the wires were cut from three different locations along the joining section.

The *T*_c_ and magnetic *J*_c_ of the Pb-Bi solders were evaluated using a PPMS from Quantum Design. For measurement of physical properties, a parallel magnetic field was applied to the length of the samples. The *T*_c_ of the solders was measured by applying a 100 Oe magnetic field and measuring the magnetic moments during cooling from 10 K to 4.2 K. The Bean model: ${J}_{c}=20(\frac{\frac{\Delta M}{V}}{a(1-\frac{a}{3b})})$ was used for estimating the *J*_c_, where *M* is the height of the *M* − *H* hysteresis loop, *a* and *b* are the sample dimensions perpendicular to the magnetic field, and *V* is the volume of the sample (*V* = *a* × *b* × *c* and *a* < b < *c*).

The transport *I*_c_(s) of the joints were measured using an American Magnetics superconducting magnet with dc current up to 200 A (where 200 A was the limit of our power supply) at 4.2 K, and magnetic fields in the range of 1.4–1.8 T, using the standard four-probe method with the criterion of 0.1 μV·cm^−1^.

A single-turn Nb-Ti closed-loop coil was fabricated to estimate the joint resistance using the field-decay measurement method. The joint fabrication process was similar to that described in Fig. 3. A Hall sensor was installed at the bottom of the coil in close vicinity to it. The coil was mounted on the test probe for the evaluation. The output voltage of the Hall sensor was measured using a voltmeter. To trap the field in the closed-loop, first, the field of the background magnet was increased to 10 kG. Subsequently, the test probe was inserted into the bore (LHe bath at 4.2 K) of the background magnet along with the coil. Following stabilization of the coil temperature and magnetic field, the background field was slowly reduced to zero. The trapped field in the coil was monitored for an extended period of time to estimate the circuit resistance. The duration between all the joints fabrication (individual joints, closed-loop, and on the prototype coil) and its characterization was about two weeks.

## Supplementary information

Supplementary Information

## Data Availability

All data generated or analyzed in this study are included in this manuscript.

## References

[1] Lvovsky Y, Stautner EW, Zhang T (2013). Novel technologies and configurations of superconducting magnets for MRI. Supercond. Sci. Technol..

[2] Thornton, R. F. Superconducting joint for superconducting wires and coils. US Patent No: 4584547 (1986).

[3] Swenson CA, Markiewicz WD (1999). Persistent joint development for high field NMR. IEEE Trans. Appl. Supercond..

[4] Cheng J (2012). Fabrication of NbTi superconducting joints for 400-MHz NMR application. IEEE Trans. Appl. Supercond..

[5] Liu S, Jiang X, Chai G, Chen J (2013). Superconducting joint and persistent current switch for a 7-T animal MRI magnet. IEEE Trans. Appl. Supercond..

[6] Motomune K, Kazutaka O, Yasunori K, Tsutomu Y, Hiroyuki W (2015). Analysis for formation of current path in the superconducting joint between Nb-Ti wires with the solder matrix replacement method. Supercond Sci Technol.

[7] Hafstrom J, Killpatrick D, Niemann R, Purcell J, Thresh H (1977). Joining NbTi superconductors by ultrasonic welding. IEEE Trans. Magne..

[8] Mizumaki S, Yamamoto A (1997). Experimental study of current sharing and transfer in superconductor joint. IEEE Trans. Appl. Supercond..

[9] Liu J, Cheng J, Wang Q (2013). Evaluation of NbTi superconducting joints for 400 MHz NMR magnet. IEEE Trans. Appl. Supercond..

[10] Phillip S, Porto JV, Parpia JM (1995). Two methods of fabricating reliable superconducting joints with multifilamentary Nb-Ti superconducting wire. J. Low Temp. Phys..

[11] Brittles GD, Mousavi T, Grovenor CRM, Aksoy C, Speller SC (2015). Persistent current joints between technological superconductors. Supercond. Sci. Technol..

[12] www.luvata.com. *Date of access: 11/06/2019*, Link, https://www.luvata.com/en/Products/Supercond.

[13] www.boltonmetalproducts.com. *Date of access: 11/06/2019*, Link, https://boltonmetalproducts.com/.

[14] Gandhi AC, Chan TS, Wu SY (2017). Phase diagram of PbBi alloys: structure-property relations and the superconducting coupling. Supercond. Sci. Technol..

[15] Campbell AM, Evetts JE, Dew-Hughes D (1968). Pinning of flux vortices in Type II superconductors. Philos. Mag. A J. Theor. Exp. Appl. Phys..

[16] Brittles, G. Persistent current joints between NbTi superconducting wires. PhD thesis, Link, https://ora.ox.ac.uk/objects/uuid:0468d27b-4d79-4ff0-a130-b9ce38b1adcb (University of Oxford, 2016).

[17] www.alfa.com. *Date of access: 11/06/2019*, Link, https://www.alfa.com/en/catalog/036641/.

[18] Patel D (2016). Evaluation of persistent-mode operation in a superconducting MgB_2_ coil in solid nitrogen. Supercond Sci Technol.

[19] Patel D (2016). A new approach to a superconducting joining process for carbon-doped MgB_2_ conductor. Supercond. Sci. Technol..

[20] Patel D (2015). MgB_2_ superconducting joints for persistent current operation. Supercond. Sci. Technol..

