# Supplementary material for: Niobium-titanium (Nb-Ti) superconducting joints for persistent-mode operation
Source: Sci Rep. 2019 Oct 3;9:14287. doi: 10.1038/s41598-019-50549-7 (PMC6776530; doi:10.1038/s41598-019-50549-7)
Supplement: Supplementary file 1 — Supplementary Information [file 41598_2019_50549_MOESM1_ESM.pdf]

Supplementary Materials

Niobium-titanium (Nb-Ti) superconducting joints for persistent-mode operation

Dipak Patel[a,b], Su-Hun Kim[a,c], Wenbin Qiu[a], Minoru Maeda[d], Akiyoshi Matsumoto[b], Gen Nishijima[b], Hiroaki Kumakura[b], Seyong Choi[d,*] & Jung Ho Kim[a,*]

[a] Institute for Superconducting and Electronic Materials, Australian Institute for Innovative Materials, University of Wollongong, North Wollongong, New South Wales 2500, Australia

[b] National Institute for Materials Science (NIMS), 1-2-1 Sengen, Tsukuba, Ibaraki 305-0047, Japan

[c] Department of Electrical Engineering, Kyungpook National University, Daegu 41566, Republic of Korea

[d] Department of Electrical Engineering, Kangwon National University, Kangwon 25913, Republic of Korea

* Correspondence and requests for materials should be addressed to S. C. (email: syc@kangwon.ac.kr) or J. H. K. (email: jhk@uow.edu.au)

Specifications	Values
Superconductor	Nb-Ti
Strand number	OK 54, Luvata
Diameter, nominal	0.90 mm
Diameter, bare	0.85 mm
Insulation	PVA, Formvar®
Number of filaments	54
Filament diameter (nominal)	77 μm
Cu/Sc ratio (nominal)	1.25
I_c at 7 T, 4.2 K	446 A

Table S1. Specifications of the Nb-Ti wire.

Pb-Bi composition (wt.%)	Solidification rate	Magnetic J_c ($A \cdot cm^{-2}$) in 1 T at 4.2 K
**In this work**		
$Pb_{44.5}Bi_{55.5}$	Commercial product[1]	2.9×10^3
$Pb_{42}Bi_{58}$		1.2×10^3
**Brittles[2]**		
$Pb_{44.5}Bi_{55.5}$	~0.29 $°C \cdot s^{-1}$	2.8×10^3
$Pb_{44.5}Bi_{55.5}$	~0.58 $°C \cdot s^{-1}$	2.8×10^3
$Pb_{60}Bi_{40}$	~0.29 $°C \cdot s^{-1}$	1.2×10^3
$Pb_{60}Bi_{40}$	~0.58 $°C \cdot s^{-1}$	0.9×10^3
$Pb_{60}Bi_{40}$	Commercial product[3]	1.2×10^3
**Motomune *et al.*[4]**		
$Pb_{60}Bi_{40}$	0.4 $°C \cdot s^{-1}$	0.8×10^3
$Pb_{60}Bi_{40}$	20 $°C \cdot s^{-1}$	0.3×10^3
$Pb_{50}Bi_{50}$	0.4 $°C \cdot s^{-1}$	1.7×10^3
$Pb_{50}Bi_{50}$	20 $°C \cdot s^{-1}$	1.7×10^3

Table S2. Comparison of magnetic J_c of various Pb-Bi alloys. The solidification rate is the rate at which the mixture solidified when it was allowed to cool down while preparing the Pb-Bi alloy for the first time.

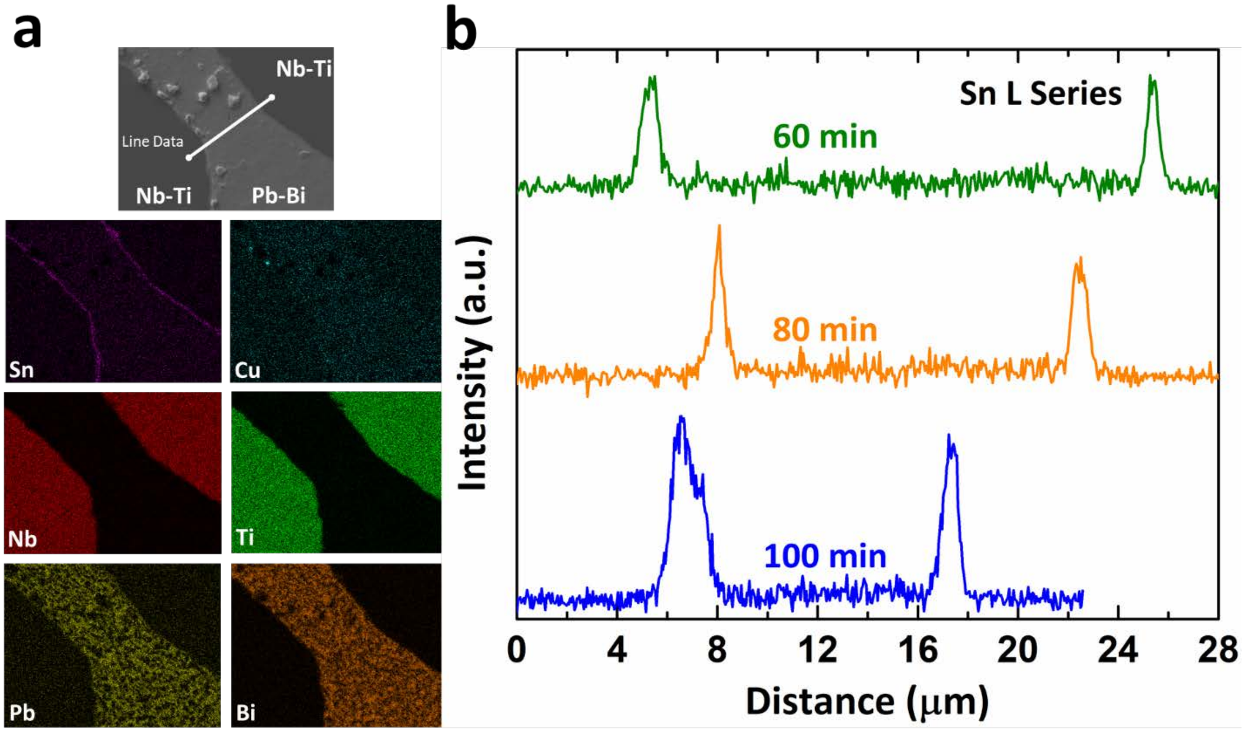

Figure S1. (a) Cross-sectional image between two filaments in the Nb-Ti wire (cutting location: bottom) after immersing in Sn for 180 min and subsequently in Pb-Bi for 60 min, and elemental maps of the cross-section, (b) Sn line maps between two Nb-Ti filaments of the samples after 60 min (see Fig. S1a.), 80 min, and 100 min of etching time in Pb-Bi (cutting location in each sample: bottom).

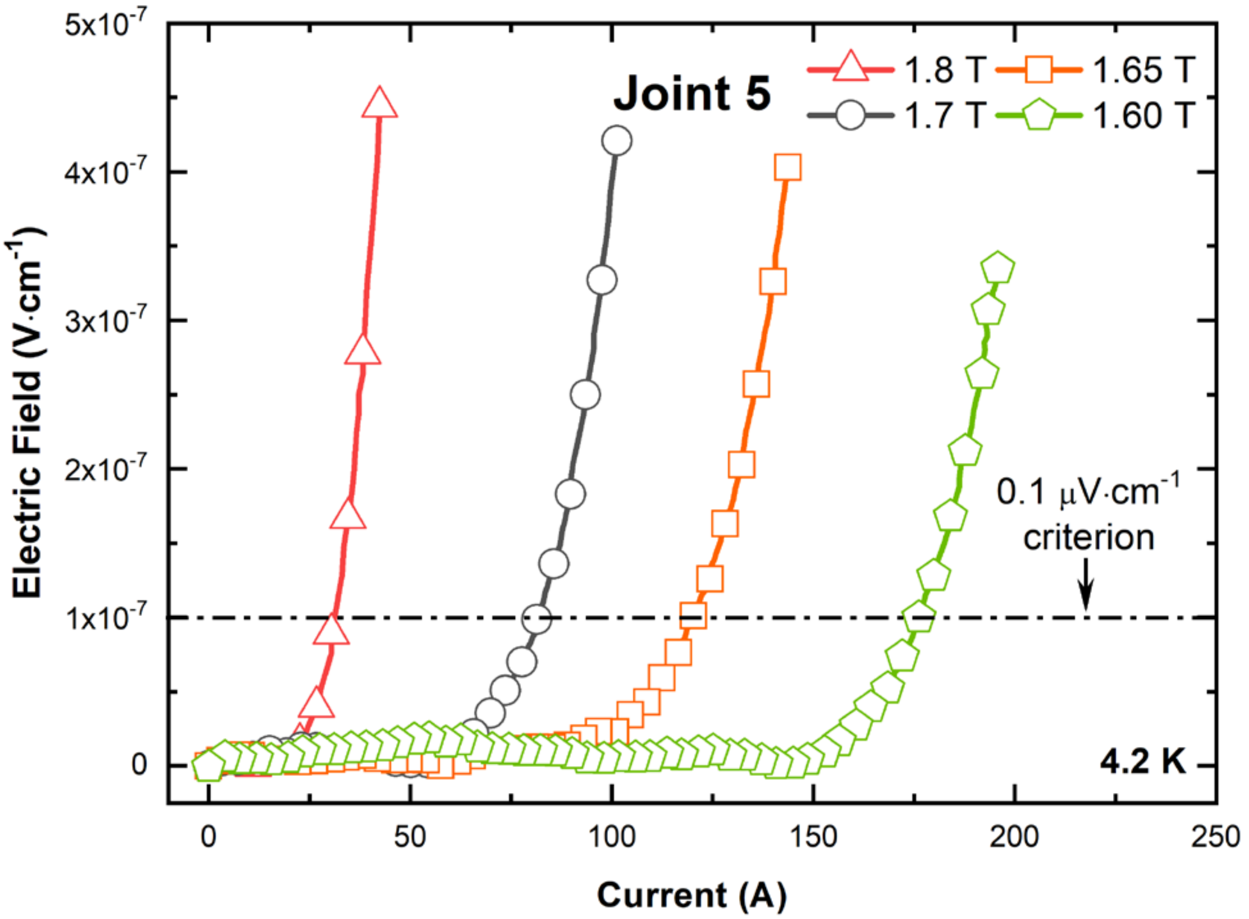

Figure S2. Electric field versus current characteristics of joint 5 in different magnetic fields at 4.2 K.

References

1 www.boltonmetalproducts.com. *Date of access: 11/06/2019*.
Link: http://www.boltonmetalproducts.com/

2 Brittles, G. *Persistent current joints between NbTi superconducting wires.* **PhD thesis**, University of Oxford (2016).
Link: https://ora.ox.ac.uk/objects/uuid:0468d27b-4d79-4ff0-a130-b9ce38b1adcb

3 www.5nplus.com. *Date of access: 11/06/2019.*
Link: https://www.5nplus.com/low-melting-point-alloys.html

4 Motomune, K., Kazutaka, O., Yasunori, K., Tsutomu, Y. & Hiroyuki, W. Analysis for formation of current path in the superconducting joint between Nb-Ti wires with the solder matrix replacement method. *Supercond. Sci. Technol.* **28**, 045019 (2015).
